# Case Report: Longitudinal multimodal imaging and microperimetry findings in PAMM: apparent capillary rarefaction and persistent sensitivity loss across 28 months

**DOI:** 10.3389/fmed.2026.1897737

**Published:** 2026-10-14

**Authors:** Qianqian Wang, Miao Du, Pengxia Wan

**Affiliations:** 1 Department of Ophthalmology, The First Affiliated Hospital, Sun Yat-Sen University, Guangzhou, Guangdong Province, China; 2 Department of Ophthalmology, The 12th People’s Hospital of Guangzhou, Guangzhou, Guangdong Province, China

**Keywords:** apparent capillary rarefaction, microperimetry, optical coherence tomography angiography, paracentral acute middle maculopathy, Proteinuria

## Abstract

**Introduction:**

Paracentral acute middle maculopathy (PAMM) is classically associated with retinal vascular occlusion or systemic vasculopathy. We describe an atypical presentation of isolated PAMM occurring in close temporal association with the abrupt withdrawal of systemic pharmacotherapy in the setting of isolated marked proteinuria, including a 28-month longitudinal analysis of structural and functional outcomes.

**Patient presentation:**

A 37-year-old male with a history of asymptomatic proteinuria presented with acute paracentral scotomas shortly after discontinuing a short course of azilsartan and hydroxychloroquine. Multimodal imaging, including spectral-domain optical coherence tomography (SD-OCT) and OCT angiography (OCTA), revealed focal inner nuclear layer (INL) infarction and deep capillary plexus (DCP) hypoperfusion, consistent with isolated PAMM in the absence of macroscopic vascular occlusion. The patient received hyperbaric oxygen therapy (HBOT) during the acute phase; a subsequent transient improvement in localized retinal sensitivity was observed, but best-corrected visual acuity (BCVA) stabilized at 20/40. However, serial microperimetry demonstrated a transient improvement in localized macular sensitivity from 3 weeks to 9 weeks post-onset, followed by a marked secondary decline from 9 weeks to 28 months. This late-stage functional loss was topographically coupled with progressive INL and outer plexiform layer (OPL) atrophy, alongside apparent capillary rarefaction on OCTA.

**Conclusions:**

Acute hemodynamic changes following medication withdrawal, combined with a potential hypercoagulability or prothrombotic risk from proteinuria, may have contributed to the development of isolated PAMM. The long-term visual prognosis is closely associated with progressive microvascular dropout and retinal atrophy. Serial microperimetry is essential to evaluate the true functional impairment in these patients.

## Background

Paracentral acute middle maculopathy (PAMM) is a distinct retinal condition characterized by ischemia of the deep capillary plexus (DCP) (1, 2). On spectral-domain optical coherence tomography (SD-OCT), the acute phase presents as a characteristic hyperreflective band at the inner nuclear layer (INL) and outer plexiform layer (OPL) junction, which subsequently progresses to permanent inner retinal atrophy. PAMM commonly occurs secondary to primary retinal vascular diseases, such as retinal vein or artery occlusions (3–5). However, it is increasingly recognized as an ocular manifestation of systemic vascular diseases or acute hemodynamic crises, including diabetes, severe hypertension, and various hypercoagulable states (6–9).

Systemic nephropathies presenting with significant proteinuria can establish a chronic hypercoagulable state due to the urinary loss of anticoagulant proteins and associated dyslipidemia (10). This environment may predispose the microcirculation to thrombotic or hypoperfusion events. When such patients experience sudden systemic blood pressure or fluid alterations—such as those following the abrupt cessation of renin-angiotensin-aldosterone system (RAAS) inhibitors or other vasoactive agents—the retinal circulation may suffer acute perfusion instability. Because the DCP is located at the termination of the retinal capillary network, it operates under lower perfusion pressure and oxygen tension, making it uniquely vulnerable to such systemic hemodynamic drops (6, 11). To date, isolated PAMM related to abrupt medication withdrawal in a patient with marked proteinuria remains rare and poorly documented.

While the acute tomographic and angiographic features of PAMM are well described, the long-term, late-stage microvascular and functional evolution remains unclear. Following acute middle retinal infarction, chronic neural atrophy occurs. This reduction in metabolic demand theoretically leads to “vascular pruning,” a process where unnecessary capillary beds regress (12–14). Optical coherence tomography angiography (OCTA) allows depth-resolved evaluation of these microvascular changes (15), but long-term longitudinal data capturing this chronic capillary remodeling in PAMM are limited. Furthermore, traditional best-corrected visual acuity (BCVA) tests often fail to reflect localized paracentral deficits (16). Fundus-controlled perimetry (microperimetry) provides precise quantification of macular sensitivity, enabling a better understanding of whether functional loss parallels chronic structural and vascular degradation (17–20).

To explore these long-term structural and functional updates, we report a 28-month follow-up of a patient who developed isolated PAMM in close temporal association with the sudden discontinuation of transient pharmacotherapy for proteinuria. We document the longitudinal clinical course, including a temporary functional improvement during the acute phase and a secondary late-stage functional decline. By correlating serial microperimetry with quantitative OCT and OCTA data, this case highlights the chronic progression of neural atrophy and apparent capillary rarefaction in late-stage PAMM.

## Case presentation

A 37-year-old Asian male presented with a two-day history of sudden-onset, painless blurred vision and a localized paracentral scotoma in his left eye. Two weeks prior to the onset of ocular symptoms, an incidental finding of proteinuria (3+) and hematuria (3+) during a routine physical examination prompted an empirical prescription of oral azilsartan medoxomil (20 mg once daily) and hydroxychloroquine sulfate (0.2 g twice daily) at an internal medicine clinic. His systemic blood pressure at that time was 138/86 mmHg. After 14 days of strict compliance, the patient abruptly discontinued both medications on his own initiative. Ocular symptoms manifested exactly two days following this cessation, accompanied by a rebound of systemic blood pressure to 140/80 mmHg. He reported no preceding flu-like illness, headaches, or neurological deficits.

Comprehensive systemic and laboratory investigations were performed concurrently to evaluate his renal, autoimmune, and vascular status. Laboratory evaluations revealed severe renal involvement, with a markedly elevated 24-hour urinary total protein of 2,392 mg/24 h (reference: 0–150 mg/24 h; representing substantial, sub-nephrotic to near-nephrotic range proteinuria according to clinical laboratory criteria) and urinary albumin of 1,196 mg/L (reference: 0-150 mg/L). Serum albumin was 44.2 g/L (reference: 40–55 g/L), serum creatinine was 73 μmol/L, eGFR was 114 mL/min/1.73 m^2^,and no systemic peripheral edema was noted clinically. Nephrology consultation was obtained, but a definitive renal biopsy was not performed; thus, the underlying primary nephropathy remains undetermined. Other notable urinalysis findings included an elevated urinary red blood cell count (3+) and an albumin-to-creatinine ratio of 77 mg/g. Serum biochemistry revealed dyslipidemia, with an elevated total cholesterol of 5.87 mmol/L and low-density lipoprotein cholesterol of 4.28 mmol/L. His glycosylated hemoglobin was mildly elevated at 6.8 mmol/L. Coagulation screening demonstrated an isolated, prolonged thrombin time of 43.2 seconds (reference: 14–21 s) with otherwise normal fibrinogen, PT, and APTT parameters. While an isolated prolonged thrombin time was observed, this laboratory finding alone is insufficient to establish a hypercoagulable state and was interpreted cautiously in the context of his proteinuria. To exclude systemic vasculitic or autoimmune disorders, an extensive laboratory workup was conducted, encompassing serum creatinine, eGFR, UREA/CREA, a complete blood count, tumor markers, thyroid function tests, rheumatologic serologies, immunoglobulins and complement levels, comprehensive metabolic panels, infectious disease screens, as well as anti-neutrophil cytoplasmic antibodies (ANCA), anti-glomerular basement membrane (anti-GBM) antibodies, antinuclear antibodies (ANA) and electrocardiography. All results were within normal limits.

This study entailed a longitudinal follow-up of the patient with PAMM over a 28-month period, with the chronological disease timeline summarized in Table 1. At the initial visit (2 days post-drugs withdrawal), the best-corrected visual acuity (BCVA) was 20/20 in the right eye and 20/200 in the left eye. Intraocular pressures were 15 mmHg bilaterally, and anterior segment examinations were unremarkable. Multimodal imaging of the left eye established the diagnosis of isolated PAMM. Color fundus photography (TOPCON, TRC0DX, IA) at day one revealed deep, wedge-shaped retinal hemorrhages radiating from the fovea, manifesting as multifocal, intraretinal “skip” lesions along the distribution of the macula (Figure 1a). Fluorescein fundus angiography (TOPCON, TRC0DX, IA) revealed disruption of the continuity of the terminal vessels temporal to the optic disc (Figure 1b). Near-infrared reflectance (NIR) imaging demonstrated hyperreflective, fern-like perivenular lesions corresponding to the ischemic zones (Figure 1e). Spectral-domain optical coherence tomography (SD-OCT, Heidelberg Spectralis OCT unit) cross-sectional scans passing through these lesions revealed well-demarcated, intensely hyperreflective, plaque-like bands confined to the inner nuclear layer (INL) and outer plexiform layer (OPL) junction, associated with focal thickening of the middle retinal layers (Figure 1e). The underlying photoreceptor inner segment/outer segment (IS/OS) junction and the retinal pigment epithelium remained morphologically intact. The central macular thickness (CMT) was increased to 300 μm, reflecting acute localized edema.

**Table 1:** Systemic and ophthalmic longitudinal data of a patient with PAMM from onset through 28 months of follow-up.

Timepoint	Systemic Interventions & Medications	Systemic Metrics (BP & Renal)	Left Eye (OS) Visual Status & Symptoms	Multimodal Ophthalmic Imaging & Key Findings
Baseline	Initiated oral Azilsartan and Hydroxychloroquine for 14 days; abrupt self-cessation of all systemic therapie.	BP: 138/86 mmHg: Proteinuria (3+)	Normal vision	—
Day 1 (PAMM onset; 2 days post-medication withdrawal)	Initiated microcirculation-optimizing therapy.	Rebound Hypertension BP: 140/80 mmHg, 24 h Urine Protein (3+); Urine occult blood: 3+; Prolonged Thrombin Time	Sudden onset: painless blurred vision and paracentral scotoma. BCVA: 20/200	Fundus/FFA: Multifocal skip hemorrhages; fern-like perivenular hyperreflectivity (Figures 1a,b)
Day 7 (Acute Presentation/HBOT Initiation)	Initiated Hyperbaric Oxygen Therapy (HBOT) (15 daily sessions)	Proteinuria (3+); Urine occult blood: 3+	Partial visual recovery: painless blurred vision & paracentral scotoma.	Isolated PAMM Diagnostic Criteria Met;
			BCVA: 20/50,	Fundus/NIR: Multifocal skip hemorrhages; fern-like perivenular hyperreflectivity (Figures 1c,e).
			Mean macular sensitivity (MS): 25.3 dB, temporal scotoma: 17–19 dB (Figure 1i).	SD-OCT: Plaque-like hyperreflective bands at INL/OPL junction; CMT: 300 μm (Figure 1e).
				OCTA: reduced 3 × 3-mm SVP VD (Figure 1g).
Weeks 3-9 (Subacute Phase/ Post-HBOT)	None (Observational follow-up).	Proteinuria, Urine occult blood: 3+	Transient Functional Improvement: BCVA: 20/40;	Fundus/NIR: Resolving multifocal skip hemorrhages.
				Microperimetry: Initial signs of localized sensitivity improvement (Figure 1o).
			MS: 27.4–28.5 dB;	SD-OCT: Resolution of middle retinal edema; early INL thinning.
			Persistent paracentral scotoma & temporal scotoma sensitivity transiently improved (11–23 dB)	OCTA: reduced 3 × 3-mm SVP VD.
Months 8–28 (Chronic Phase)^a^	None (Observational follow-up).	Normal	Permanent Impairment: BCVA stabilized at 20/40;	Neural Atrophy & Capillary Rarefaction:
				Fundus: Complete hemorrhage resolution (Figure 1d).
			MS: 28.5 dB. Fixed, absolute paracentral scotoma.	SD-OCT: Severe permanent INL atrophy and OPL thinning; CMT down to 267 μm (Figure 1f).
				OCTA: Late-stage microvascular dropout; 3 × 3-mm (19.9 mm^−1^) SVP VD decreased (Figure 1h). Capillary rarefaction (Figures 1k,l,n)
				Microperimetry: Secondary, progressive late-stage functional decline. (Figures 1j,o).

a Note: Intermediate observational visits were conducted at months 8, showing stabilization of structural atrophy; the 28-month endpoint reflects the final follow-up data presented in text and figures.

**Figure 1:**
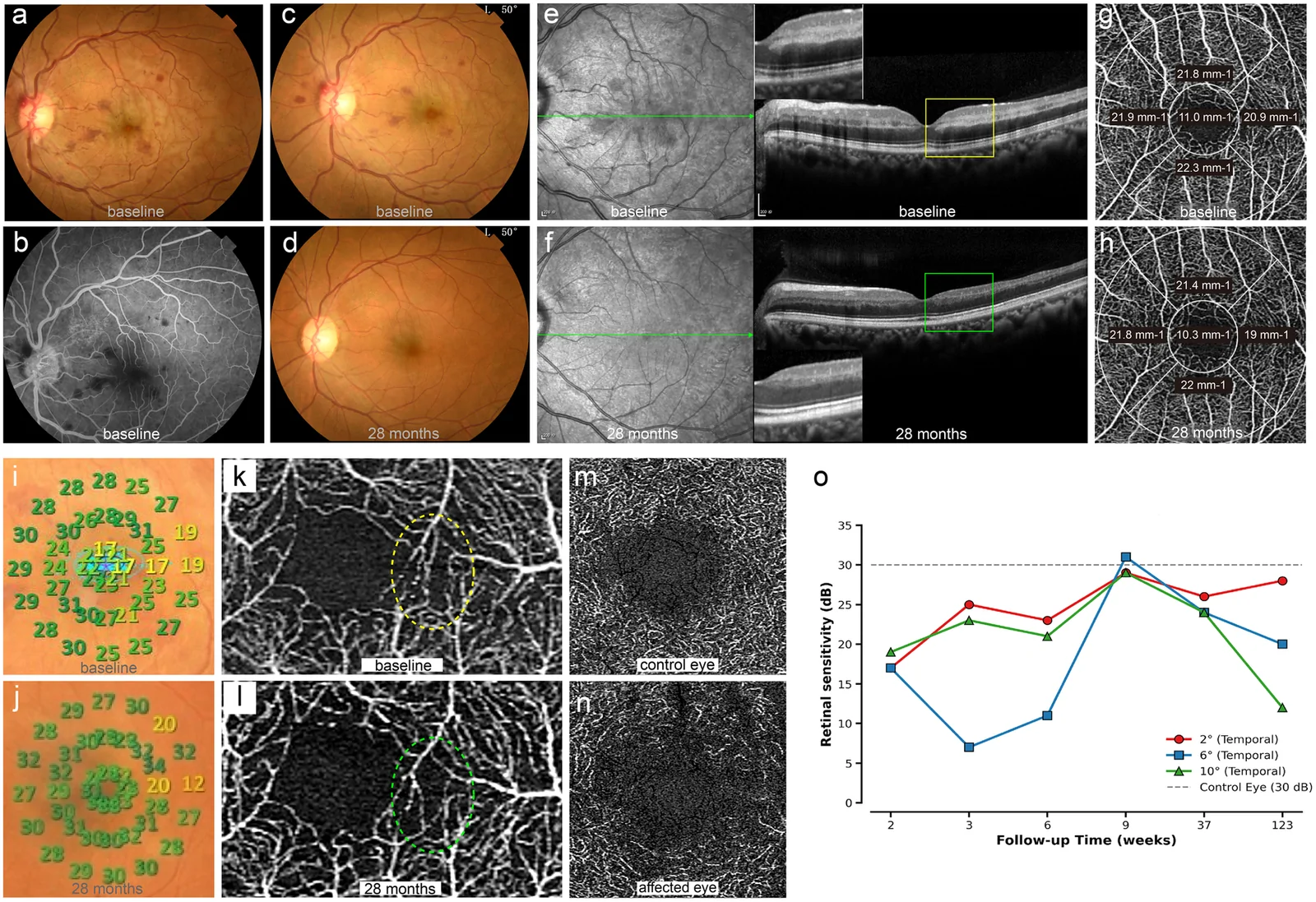
Multimodal imaging demonstrating the longitudinal structural and functional evolution of paracentral acute middle maculopathy (PAMM) over a 28-month follow-up. **(a,b)** Fluorescein fundus angiography (FFA) showed radial Henle fiber layer hemorrhage and fluorescence shielding that was consistent with the lesion locations observed in CFP. **(c,d)** Color fundus photography at baseline **(c)** and 28 months **(d)**, showing the initial multifocal “skip” lesions and their subsequent resolution. **(e,f)** Near-infrared reflectance and corresponding spectral-domain optical coherence tomography (SD-OCT) B-scans. Baseline SD-OCT **(e)** reveals a characteristic plaque-like hyperreflective band at the inner nuclear layer (INL) and outer plexiform layer (OPL) junction, as shown in yellow box. At 28 months **(f)**, marked INL thinning and OPL attenuation are observed, indicating permanent neural atrophy, as shown in green box. **(g,h)** Optical coherence tomography angiography (OCTA) of the superficial vascular plexus (SVP). Compared to baseline **(g)**, the 28-month follow-up **(h)** demonstrates a progressive reduction in vessel density at temporal side of fovea, corresponding to late-stage capillary rarefaction. **(i,j)** Fundus-controlled microperimetry maps at baseline **(i)** and 28 months **(j)**, illustrating the topographic distribution of localized retinal sensitivity (dB). Magnified OCTA en face images at the SVP **(k,l)** of the temporal regions in the affected eye at baseline (yellow circle) and at 28 months (green circle), and at the deep vascular plexus (DVP) of the control eye **(m)** and the affected eye **(n)**, detailing the progressive microvascular remodeling and capillary rarefaction from the acute phase to the final follow-up. **(o)** A longitudinal line graph tracking localized retinal sensitivity across the temporal side of the fovea in three concentric temporal zones (2°, 6°, and 10°) over 123 weeks. The graph highlights an initial transient functional recovery during the subacute phase, succeeded by a marked secondary functional decline, contrasting with the stable sensitivity of the unaffected control eye (dashed line).

Optical coherence tomography angiography (OCTA) was acquired using the AngioPlex Cirrus 5000 HD-OCT system (Carl Zeiss Meditec, Dublin, CA) over 3 × 3-mm and 6 × 6-mm scan patterns centered on the fovea. Only scans with a signal strength = 10/10 without significant motion or shadow artifacts were included for quantitative analysis. Automated segmentation boundaries were set by the device software: the superficial vascular plexus (SVP/SVC) was segmented from the internal limiting membrane (ILM) to the inner plexiform layer (IPL), and the deep capillary plexus (DCP/DVC/DVP) was segmented from the inner nuclear layer (INL) to the outer plexiform layer (OPL). Superficial vessel density (VD, mm^−1^) was automatically calculated by the built-in software as the total length of perfused vasculature divided by the scan area. Quantitative assessment showed that the mean vessel density (VD) of the superficial vascular plexus (SVP) within the 3 × 3-mm area was reduced in the affected left eye compared to the unaffected fellow eye (20.5 vs. 22.5 mm^−1^) (Figure 1g), whereas the VD within the expanded 6 × 6-mm region was initially comparable between the two eyes (18.9 vs. 18.7 mm^−1^). En face DVP images demonstrated localized signal void/hypoperfusion in the area corresponding to the INL hyperreflectivity; however, due to residual projection artifacts and severe middle retinal structural alterations in late stages, qualitative DVP assessment is shown (Figures 1m,n) while quantitative metrics focused on the SVP. Fundus-controlled microperimetry (Nidek-MP3, NAVIS-EX 1.8.0, Nidek Co., Ltd., Gamagori, Japan) was performed using a standardized 40-point radial grid centered on the fovea, Goldmann III stimulus size, stimulus duration of 200 ms, and background luminance of 31.4 apostilbs (4 asb/10 cd/m^2^). A 4-2 threshold strategy was applied. Fixation stability was recorded automatically, and tests with false-positive or false-negative rates >15% were deemed unreliable and excluded. To ensure precise topographic comparison across follow-ups, auto-tracking eye registration was utilized to re-test the exact same retinal loci. Microperimetry quantified a depressed baseline mean macular sensitivity (MS) of 25.3 dB in the left eye compared to 30.8 dB in the right eye, with topographic mapping showing localized scotomas (17-19 dB) clustered densely in the temporal parafoveal region (Figure 1i).

Systemic therapy aimed at optimizing microcirculation (including oral neurotrophic and vasoactive agents) was initiated immediately. One week after symptom onset, hyperbaric oxygen therapy [HBOT; 2.0 atmospheres absolute (ATA), 100% O_2_, 60 minutes daily] was introduced as an adjunctive intervention and administered once daily for 15 consecutive sessions until week 4.

The patient was monitored using serial multimodal evaluations over a 28-month follow-up period. At the final 28-month visit, the BCVA in the left eye stabilized at 20/40, though the patient complained of a persistent, fixed paracentral scotoma. Serial color fundus photography documented complete resolution of the intraretinal hemorrhages and a return to a normal fundus appearance (Figure 1d). However, longitudinal SD-OCT demonstrated progression from acute hyperreflectivity to irreversible tissue loss, characterized by severe thinning of the INL and marked attenuation of the OPL in the regions previously corresponding to the acute lesions (Figure 1f). CMT decreased to 267 μm, indicating the resolution of edema and the onset of intraretinal atrophy.

Longitudinal OCTA confirmed a permanent reduction in microvascular parameters. In the 3 × 3-mm scan area, the mean SVP VD remained decreased at 19.9 mm^−1^ compared to 22.5 mm^−1^ in the fellow eye (Figure 1h), confirming late-stage microvascular dropout consistent with apparent capillary rarefaction (Figures 1k,l). In the 6 × 6-mm area, which was normal at baseline, the mean SVP VD ultimately exhibited a delayed reduction (17.6 vs.18.6 mm^−1^).

Serial microperimetry unmasked a distinct structure-function dissociation. The global mean MS improved from the baseline value of 25.3 dB to 28.5 dB at 28 months, yet failed to achieve the status of the healthy control eye (30.8 dB) (Figure 1j). Topographic analysis of the temporal foveal region demonstrated a biphasic functional response: a transient, marked improvement in localized retinal sensitivity was recorded between weeks 3 and 9 post-onset, followed by a secondary, progressive decline from week 9 through the final 28-month follow-up (Figure 1o). This late-stage secondary functional decline topographically co-localized with the areas of progressive retinal layer atrophy and capillary dropout identified on SD-OCT and OCTA.

## Discussion and conclusion

This longitudinal case study monitored the 28-month clinical course of a patient with isolated paracentral acute middle maculopathy (PAMM), topographically correlating retinal microstructure and microvasculature changes with visual function via multimodal imaging. The acute phase of PAMM is characterized by a distinctive hyperreflective band at the inner nuclear layer (INL) and outer plexiform layer (OPL) junction on spectral-domain optical coherence tomography (SD-OCT), which topographically co-localized with an acute reduction in localized macular sensitivity (MS) measured by microperimetry. This observation is consistent with previous literature describing early paracentral scotomas resulting from deep capillary plexus (DCP) ischemia (1, 15). The acute inner retinal edema within the macular region may induce light scattering and mechanical impairment, contributing to the initial functional deficit (20, 21). During the chronic stage, the resolution of middle retinal edema gives way to permanent thinning of the INL and attenuation of the OPL. Our findings suggest that despite a partial recovery of gross visual acuity, the chronic loss of middle retinal neurons and structural remodeling can result in permanent localized functional deficits. This structure-function relationship aligns with longitudinal observations documented in other macular diseases, including age-related macular degeneration (18, 22), epiretinal membranes (20), and retinal dystrophies (19).

The temporal relationship between the onset of symptoms and the abrupt cessation of systemic azilsartan medoxomil and hydroxychloroquine sulfate introduces a compelling clinical scenario. Rather than a classical thromboembolic event, this presentation raises the possibility that acute systemic hemodynamic fluctuations may serve as a potential trigger for PAMM. Renin-angiotensin-aldosterone system (RAAS) inhibitors, such as azilsartan, play a critical role in regulating systemic blood pressure and local tissue perfusion (23). The sudden withdrawal of such medication can provoke a rebound increase in systemic blood pressure or an acute disturbance in local microvascular autoregulation. In this patient, the underlying state associated with marked proteinuria (2,392 mg/24 h) and concomitant dyslipidemia likely heightened the vulnerability of the retinal microcirculation.

Given the deep, wedge-shaped intraretinal hemorrhages and multifocal “skip” lesions observed at presentation, several differential diagnoses must be carefully considered. First, a non-ischemic branch retinal vein occlusion (BRVO) can present with localized intraretinal hemorrhages and secondary DCP hypoperfusion (24). However, the absence of optic disc edema, generalized venous tortuosity, or engorgement, combined with the classic plaque-like hyperreflectivity restricted to the INL/OPL junction, rendered primary BRVO less likely. Second, acute macular neuroretinopathy (AMN) represents a key differential diagnosis, as it shares a similar ischemic spectrum with PAMM (25). AMN was excluded because the hyperreflective bands on SD-OCT were confined to the middle retina (INL) rather than the outer retinal layers (outer nuclear layer and ellipsoid zone), which typically characterize AMN (1, 26). Third, given the patient's severe proteinuria, systemic autoimmune or connective tissue diseases (such as systemic lupus erythematosus or antiphospholipid syndrome) complicated by lupus retinopathy or microthrombosis were considered (27). However, the comprehensive and entirely negative autoimmune panel (including ANA, ANCA, and anti-GBM antibodies) argued against an active systemic vasculitic or autoimmune etiology. Therefore, the overall clinical and imaging findings supported a diagnosis of isolated PAMM, with systemic hemodynamic and renal/prothrombotic factors considered as possible contributors.

The unique vulnerability of the DCP to such hemodynamic shifts can be explained by its vascular anatomy (14, 28). The retinal capillary network is predominantly arranged in a serial configuration, wherein arterial inflow supplies the superficial vascular complex (SVC) and subsequently drains into the deep vascular complex (DVC). Positioned at the terminus of this oxygenation gradient, the DVC operates under lower perfusion pressure and lower oxygen tension. Consequently, even a transient systemic hemodynamic fluctuation or a localized reduction in perfusion pressure can selectively compromise the DCP, leading to focal middle retinal infarction without macroscopic large-vessel occlusion. HBOT has been investigated as an adjunctive treatment for various retinal ischemic conditions (29, 30), including central retinal artery occlusion (CRAO) and branch retinal artery occlusion (BRAO), based on its capacity to increase oxygen tension in hypoxic tissues and reduce ischemia-induced edema. However, the evidence for its efficacy in isolated PAMM remains limited, and randomized controlled trials are lacking. The patient received HBOT during the acute/subacute phase. A subsequent transient improvement in localized retinal sensitivity was observed, but the single-case observational design precludes any inference regarding treatment efficacy. While it could be hypothesized that HBOT temporarily mitigated cellular hypoxia within an ischemic penumbra, the subsequent functional decline is consistent with the possibility that acute interventions did not prevent the longer-term structural and functional sequelae once irreversible ischemic injury was established.

The implementation of longitudinal optical coherence tomography angiography (OCTA) provided valuable insights into the chronic microvascular dynamics (14). In the acute phase, ischemia and subsequent intracellular edema may elevate local tissue pressure, increasing resistance to microvascular flow. This is reflected by the acute reduction in superficial vascular plexus (SVP) vessel density (VD) within the central 3 × 3-mm area. Over the 28-month follow-up, the primary necrotic involution of the middle retinal neurons significantly lowered the local metabolic demand. Consequently, the corresponding capillary beds underwent secondary regression—a physiological adaptation recognized as apparent capillary rarefaction. Although PAMM primary pathology resides in the DCP, the observed long-term SVP vessel-density reduction reflects secondary retrograde capillary dropout or global capillary network remodeling following severe middle retinal atrophy. The late-stage, delayed reduction in VD observed within the 6 × 6-mm area further supports the progressive nature of this microvascular remodeling.

A notable finding in this case was the biphasic functional response captured by serial microperimetry. The initial transient improvement in localized retinal sensitivity between weeks 3 and 9 post-onset was followed by a secondary, progressive functional decline from week 9 to month 28. This secondary decline topographically co-localized with the permanent neural atrophy and microvascular dropout seen on SD-OCT and OCTA. This structure-function dissociation highlights that BCVA may not fully reflect the true degree of permanent paracentral visual disability in these patients.

The principal strength of this report lies in its comprehensive, long-term multimodal dataset, which offers a detailed anatomical and functional timeline of PAMM progression. Nevertheless, several limitations must be acknowledged. As a single case report, the observed clinical course and the chronological association with medication withdrawal cannot establish definitive causal links or generalizable clinical rules. The patient was empirically prescribed azilsartan and hydroxychloroquine following an incidental finding of proteinuria and hematuria; a definitive renal biopsy was not performed, leaving the primary nephropathy undetermined. Furthermore, the observational nature of this report precludes any conclusions regarding HBOT efficacy. Prospective, large-scale cohort studies are required to further elucidate the precise systemic risk factors and optimal management strategies for isolated PAMM.

In conclusion, this 28-month follow-up suggests that isolated PAMM may be temporally associated with acute systemic hemodynamic fluctuations, particularly when exacerbated by an underlying prothrombotic state such as proteinuria. While a transient functional improvement was observed during the acute/subacute period following HBOT, no conclusions regarding treatment efficacy can be drawn. The long-term visual outcome appears closely associated with irreversible middle retinal atrophy and secondary apparent capillary rarefaction. These findings underscore the importance of integrating microperimetry and detailed longitudinal OCTA into the clinical assessment of retinal vascular diseases.

## Data Availability

The original contributions presented in the study are included in the article/Supplementary Material, further inquiries can be directed to the corresponding author.

## References

[1] Sarraf D Rahimy E Fawzi AA Sohn E Barbazetto I Zacks DN. Paracentral acute middle maculopathy: a new variant of acute macular neuroretinopathy associated with retinal capillary ischemia. JAMA Ophthalmol. (2013) 131(10):1275–87. 10.1001/jamaophthalmol.2013.4056 23929382

[2] Dansingani KK Freund KB. Paracentral acute middle maculopathy and acute macular neuroretinopathy: related and distinct entities. Am J Ophthalmol. (2015) 160(1):1–3.e2. 10.1016/j.ajo.2015.05.001 26054463

[3] Zhao PY Johnson MW McDonald HR Sarraf D. Paracentral acute middle maculopathy and the ischemic cascade: toward interventional management. Am J Ophthalmol. (2022) 234:15–19. 10.1016/j.ajo.2021.07.030 34343488

[4] Samanta R Dogra M Puthalath AS Sood G. Paracentral acute middle maculopathy with occlusive retinal vasculitis in presumed intraocular tuberculosis and hyperhomocysteinemia. BMJ Case Rep. (2022) 15(11):e249327. 10.1136/bcr-2022-249327 36368732 PMC9660497

[5] Chen X Rahimy E Sergott RC Nunes RP Souza EC Choudhry N. Spectrum of retinal vascular diseases associated with paracentral acute middle maculopathy. Am J Ophthalmol. (2015) 160(1):26–34.e1. 10.1016/j.ajo.2015.04.004 25849522

[6] Scharf J Freund KB Sadda SV Sarraf D. Paracentral acute middle maculopathy and the organization of the retinal capillary plexuses. Prog Retin Eye Res. (2021) 81:100884. 10.1016/j.preteyeres.2020.100884 32783959

[7] Abtahi S-H Nourinia R Mazloumi M Nouri H Arevalo JF Ahmadieh H. Retinal ischemic cascade: new insights into the pathophysiology and imaging findings. Surv Ophthalmol. (2023) 68(3):380–387. 10.1016/j.survophthal.2022.11.009 36464134

[8] Limoli C Raja LD Wagner SK Ferraz D Bolz M Vujosevic S. Exploring patient demographics and presence of retinal vascular disease in paracentral acute middle maculopathy. Am J Ophthalmol. (2024) 260:182–189. 10.1016/j.ajo.2023.12.010 38104758

[9] Limoli C Raja LD Wagner SK Patel PJ Nicholson L Bolz M. Paracentral acute middle maculopathy and risk of cardiovascular disease, stroke, and death: a longitudinal study. Am J Ophthalmol. (2024) 267:286–292. 10.1016/j.ajo.2024.08.005 39154925

[10] Gamez M Ramnath RD Butler MJ Foster RR Satchell SC. The glomerular endothelial glycocalyx as a therapeutic target in proteinuric kidney disease. Nat Rev Nephrol. (2026) 22(3):229–241. 10.1038/s41581-025-01028-y 41272309

[11] Selvam S Kumar T Fruttiger M. Retinal vasculature development in health and disease. Prog Retin Eye Res. (2018) 63:1–19. 10.1016/j.preteyeres.2017.11.001 29129724

[12] Santamaría R González-Álvarez M Delgado R Esteban S Arroyo AG. Remodeling of the microvasculature: may the blood flow be with you. Front Physiol. (2020) 11:586852. 10.3389/fphys.2020.586852 PMC7593767 33178049

[13] Fumi D Ruggeri F Fasciolo D Antonello E Burtini G Abdolrahimzadeh S. Paracentral acute middle maculopathy (PAMM) in ocular vascular diseases-what we know and future perspectives. Vision (Basel). (2025) 9(1):19. 10.3390/vision9010019 40137931 PMC11946784

[14] Korn C Augustin HG. Mechanisms of vessel pruning and regression. Dev Cell. (2015) 34(1):5–17. 10.1016/j.devcel.2015.06.004 26151903

[15] Nemiroff J Kuehlewein L Rahimy E Tsui I Doshi R Gaudric A. Assessing deep retinal capillary ischemia in paracentral acute middle maculopathy by optical coherence tomography angiography. Am J Ophthalmol. (2016) 162:121–132.e1. 10.1016/j.ajo.2015.10.026 26562176

[16] Ríos HA Lövestam-Adrian M Plainis S Tsilimbaris M Joussen AM Keegan D. Additional measures of macular function beyond visual acuity. Graefes Arch Clin Exp Ophthalmol. (2024) 262(6):1723–1736. 10.1007/s00417-023-06272-1 PMC11106142 37938378

[17] Pfau M Jolly JK Wu Z Denniss J Lad EM Guymer RH. Fundus-controlled perimetry (microperimetry): application as outcome measure in clinical trials. Prog Retin Eye Res. (2021) 82:100907. 10.1016/j.preteyeres.2020.100907 33022378 PMC12872260

[18] Goh KL Chen FK Balaratnasingam C Abbott CJ Hodgson LAB Guymer RH. Cuticular drusen in age-related macular degeneration: association with progression and impact on visual sensitivity. Ophthalmology. (2022) 129(6):653–660. 10.1016/j.ophtha.2022.01.028 35120992

[19] Lad EM Duncan JL Liang W Maguire MG Ayala AR Audo I. Baseline microperimetry and OCT in the RUSH2A study: structure-function association and correlation with disease severity. Am J Ophthalmol. (2022) 244:98–116. 10.1016/j.ajo.2022.08.013 36007554 PMC9712171

[20] Wang Q Wang C Su Y Yu F Chen T Dong X. Topographic correlation of microperimetry with foveal microstructure characteristics in idiopathic epiretinal membrane patients with an ectopic inner foveal layer. Eye Vis (Lond. (2025) 12(1):4. 10.1186/s40662-024-00419-9 39891311 PMC11786330

[21] Bringmann A Syrbe S Görner K Kacza J Francke M Wiedemann P. The primate fovea: structure, function and development. Prog Retin Eye Res. (2018) 66:49–84. 10.1016/j.preteyeres.2018.03.006 29609042

[22] Goh KL Kumar H Hadoux X Jannaud M Abbott C Hodgson L. Hyperpigmentary abnormalities in age-related macular degeneration: association with progression and impact on visual sensitivity. Br J Ophthalmol. (2024) 108(2):263–267. 10.1136/bjo-2022-322676 36564147

[23] Zhu L Wei G-C Xiao Q Chen Q-L Zhao Q Li X- Efficacy and safety of azilsartan medoxomil in the treatment of hypertension: a systematic review and meta-analysis. Front Cardiovasc Med. (2024) 11:1383217. 10.3389/fcvm.2024.1383217 39026999 PMC11254823

[24] Hayreh SS. Photocoagulation for retinal vein occlusion. Prog Retin Eye Res. (2021) 85:100964. 10.1016/j.preteyeres.2021.100964 33713810

[25] Iovino C Au A Ramtohul P Bacci T AlBahlal A Khan AM. Coincident PAMM and AMN and insights into a common pathophysiology. Am J Ophthalmol. (2022) 236:136–146. 10.1016/j.ajo.2021.07.004 34283978

[26] Dinh RH Tsui E Wieder MS Barash A Park MM Rahimy E. Acute macular neuroretinopathy and coronavirus disease 2019. Ophthalmol Retina. (2023) 7(2):198–200. 10.1016/j.oret.2022.09.005 36216223 PMC9546455

[27] Wakabayashi T Hara C Nishida K. Retinal arteriogenesis after vaso-occlusive lupus retinopathy. Ophthalmology. (2022) 129(10):1191. 10.1016/j.ophtha.2022.02.030 35753862

[28] Fruttiger M. Development of the retinal vasculature. Angiogenesis. (2007) 10(2):77–88. 10.1007/s10456-007-9065-1 17322966

[29] Chiabo J Kauert A Casolla B Contenti J Nahon-Esteve S Baillif S. Efficacy and safety of hyperbaric oxygen therapy monitored by fluorescein angiography in patients with retinal artery occlusion. Br J Ophthalmol. (2024) 108(7):956–962. 10.1136/bjo-2023-323972 37722767 PMC11228221

[30] Oguz H Sobaci G. The use of hyperbaric oxygen therapy in ophthalmology. Surv Ophthalmol. (2008) 53(2):112–20. 10.1016/j.survophthal.2007.12.002 18348877

